# Photodynamic therapy of extrahepatic cholangiocarcinoma using digital cholangioscopy

**DOI:** 10.1590/0102-672020190001e1490

**Published:** 2020-11-13

**Authors:** Vítor Ottoboni BRUNALDI, José Eduardo BRUNALDI, José Dirceu VOLLET-FILHO, Mariangela Ottoboni BRUNALDI, José Celso ARDENGH, Vanderlei Salvador BAGNATO, José Sebastião DOS-SANTOS, Rafael KEMP

**Affiliations:** 1 Ribeirão Preto Medical School, University of São Paulo, Center for Gastrointestinal Endoscopy, Department of Surgery and Anatomy, Ribeirão Preto, SP, Brazil; 2 University of São Paulo, Institute of Physics of São Carlos, São Carlos, SP, Brazil; 3 Ribeirão Preto Medical School, University of São Paulo, Pathology Department, Ribeirão Preto, SP, Brazil

**Keywords:** Endoscopy, Cholangiocarcinoma, Jaundice, Cholangiopancreatography, endoscopic retrograde, Endoscopia, Colangiocarcinoma, Icterícia, CPRE

## Abstract

**Background:**

Cholangiocarcinoma is an aggressive neoplasm that usually requires palliative biliary drainage. Photodynamic therapy (PDT) has been described as a successful adjunct treatment to malignant biliary obstruction.

**Aim:**

To describe the use of digital cholangioscope to help provide laser light during biliary PDT session using locally developed light source.

**Method:**

Patient receives intravenous photosensitizer 24 h before the procedure. It starts with a regular duodenoscopy. After identification of the major papilla and retrograde cannulation, the digital cholangioscope is introduced into the common bile duct. Then, the cholangioscopic examination helps to identify the neoplastic stricture. Under direct visualization lighting catheter is advanced through the cholangioscope. Repositioning is recommended every centimeter to cover all strictured area. At the end of the procedure, a final cholangioscopy assesses the bile duct for the immediate result and adverse events.

**Result:**

This procedure was applied in one 82-year-old male due to obstructive jaundice in the last two months. EUS and ERCP revealed a severe dilation of the common bile duct associated with choledocholithiasis. Besides, was revealed dilation of hepatic duct up to a well-circumscribed hypoechoic solid mass measuring 1.8x2 cm compressing the common hepatic duct. The mass was deemed unresectable and the patient was referred for palliative treatment with PDT. He remained asymptomatic for three months. He perished due to complications 15 months after the PDT session.

**Conclusion:**

Digital cholangioscopy-guided biliary PDT is feasible and seems safe and effective as an adjunct modality in the palliation of extrahepatic cholangiocarcinoma.

## Graphical Abstract

[Fig f1a]

**Figure f1a:**
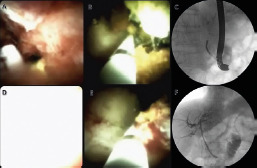
Photodynamic therapy (PDT) of an extrahepatic cholangiocarcinoma using a digital cholangioscopy to deliver the laser

## RESUMO

**Racional:**

Colangiocarcinoma é neoplasia agressiva que geralmente exige drenagem biliar paliativa. A terapia fotodinâmica (TFD) tem sido descrita como tratamento adjunto bem-sucedido para tratar obstrução biliar maligna*.*

**Objetivo:**

Descrever o emprego do colangioscópio digital para ajudar a fornecer luz de laser durante sessão de TFD biliar usando fonte de luz desenvolvida localmente.

**Método:**

Paciente recebe fotossensibilizador intravenoso 24 h antes do procedimento que começa com duodenoscopia regular. Após a identificação da papila principal e da canulação retrógrada, o colangioscópio digital é introduzido no ducto biliar comum. Em seguida, o exame colangioscópico ajuda a identificar a estenose neoplásica. Sob visualização direta, o cateter de iluminação avança através do colangioscópio. Reposicionamento é feito a cada centímetro. Ao final colangioscopia avalia o ducto biliar quanto ao resultado imediato e a eventos adversos.

**Resultado:**

Este procedimento foi aplicado em um homem de 82 anos devido à icterícia obstrutiva nos últimos dois meses. EUS e CPRE revelaram dilatação grave do ducto biliar comum associada à coledocolitíase. Além disso, havia dilatação do ducto hepático até massa sólida hipoecóica bem circunscrita, medindo 1,8x2 cm, comprimindo o ducto hepático comum. Ela foi considerada irressecável e paciente encaminhado para tratamento paliativo com TFD que permaneceu assintomático por três meses. Morreu devido a complicações 15 meses após a sessão de TFD.

**Conclusão:**

A TFD biliar guiada por colangioscopia digital é viável e parece segura e eficaz como modalidade auxiliar na paliação de colangiocarcinoma extra-hepático.

## Graphical Abstract

[Fig f1as]

**Figure f1as:**
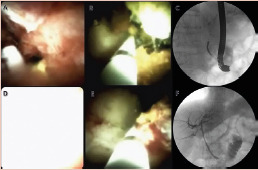
Terapia fotodinâmica (TFD) de colangiocarcinoma extra-hepático utilizando colangioscopia digital para fornecer o laser

## INTRODUCTION

The cholangiocarcinoma (CC) is a rare but aggressive neoplasm originating from the biliary epithelial cells^2^ ^,^ ^4^. Its treatment is complex and multidisciplinary^5^ but surgical resection is still the only curative option. Unfortunately, most cases present with unresectable masses at diagnosis^4^ ^,^ ^6^ ^,^ ^14^.

The non-curative systemic treatment involves chemotherapy, plus radiation therapy in selected cases. Symptomatic relief is central as well, that is, optimized clinical support. The extrahepatic CC typically presents with obstructive jaundice^12^, rendering biliary drainage also necessary. Besides relieving symptoms and preventing cholangitis, drainage is also mandatory prior to the beginning of systemic chemotherapy^1^.

The endoscopic retrograde cholangiopancreatography (ERCP) is the preferred route for biliary drainage in the palliative setting^12^. It involves the removal of debris and biliary stent placement, using a self-expandable metallic stent. Local ablative methods such as radiofrequency and photodynamic therapy (PDT) may be also associated^16^.

The PDT is a neoplastic-specific laser-based modality that has been introduced as adjunctive therapy to biliary stenting. It requires a previous intravenous infusion of a photosensitizing agent that concentrates in neoplastic cells. During the PDT session, the tumor is exposed to a specific wavelength of light that interacts with the photosensitizer. Such interaction creates highly cytotoxic oxygen-free radicals thus leading to the destruction of tumor cells while sparing healthy ones^3^ ^,^ ^8^.

Recently, the single-operator cholangioscopy has been described in helping deliver light for biliary PDT. Some authors advocate it allows precise targeting, hypothetically improving outcomes^10^ ^,^ ^15^. However, those are scattered reports and most describe the use of the first-generation cholangioscope. 

The aim of this paper is to describe the use of a digital cholangioscope to direct lighting during a PDT session in extrahepatic CC using a locally-developed laser source.

## METHOD

### Technique

#### PDT procedure information

**FIGURE 1:**
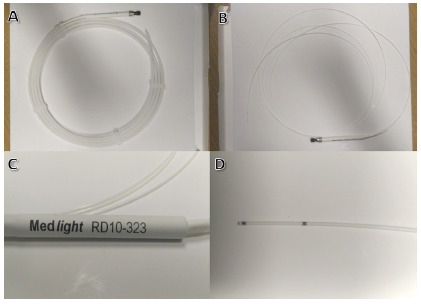
A) Fiber with enclosure; B) fiber without enclosure; C) proximal end of the lighting catheter; D) distal end of the fiber and between the two metal marks is the 1 cm laser diffuser

**FIGURE 2:**
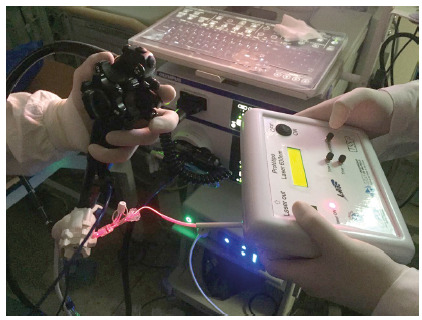
The handheld controller of the light source

The patient receives an intravenous photosensitizer (Photogen^®^, King of Prussia, PA, USA - 1.5 mg/kg) 24 h before the procedure. Its peak of light absorption is at 630 nm wavelength. The procedure starts with a regular duodenoscopy (Olympus TJF-180) under general anesthesia. After identification of the major papilla and retrograde cannulation, the digital cholangioscope (SpyGlass^TM^ DS, Boston Scientific, Natick, MA) is introduced into the common bile duct. Then, the cholangioscopic examination helps to identify the neoplastic stricture. Under direct visualization, the lighting catheter (Medlight S.A., RD10-323, Switzerland) is advanced through the cholangioscope. It consists of a typical 3-way ERCP cannula. The first port carries a 1 cm-long cylindrical light diffuser at its end. Two black radiopaque marks demarcate the limits of the diffuser. The second port accommodates a 0.025-inch guidewire and the third is an injection port (Figure 1). After positioning under cholangioscopic guidance, the lighting initiates. The dose is 90 J/cm² with a power of 70 mW/cm² (Figure 2). Repositioning is recommended every centimeter to cover all strictured area. At the end of the procedure, a final cholangioscopy assesses the bile duct for the immediate result and adverse events.

#### Post-procedure care

The patient is kept fasting for the following 24 h. If no adverse event is detected, the oral diet is initiated. Discharge is done under strict orientation on photoprotection (avoidance of light exposure and wearing sunglasses), especially during the first week after the PDT session.

## RESULTS

This procedure was applied in one 82-year-old male referred to our tertiary center due to obstructive jaundice and weight loss in the last two months. An endoscopic ultrasound (EUS) revealed a severe dilation of the common bile duct associated with choledocholithiasis. The ERCP confirmed those findings but was not able to remove the stones due to disproportionate dilatation of the common bile duct. Therefore, we opted for biliary plastic stenting. The patient experienced improvement in his clinical condition but the cholestasis recurred 45 days after the first procedure. Two further ERCPs failed to remove the stones. During his third procedure, a long irregular stricture of the common hepatic duct became clear and he was referred to another sonographic evaluation.

Besides choledocholithiasis, the new EUS revealed dilation of hepatic duct up to a well-circumscribed hypoechoic solid mass measuring 1.8x2 cm compressing the common hepatic duct. The mass seemed to involve the common hepatic artery as well. The EUS-guided fine-needle aspiration confirmed the diagnosis of moderately differentiated cholangiocarcinoma (Bismuth I).

**FIGURE 3:**
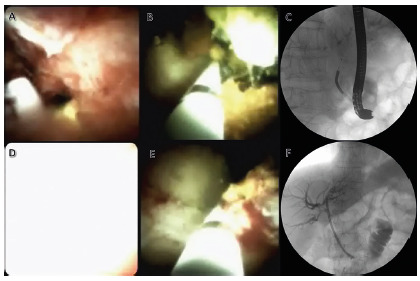
Photodynamic therapy (PDT) of an extrahepatic cholangiocarcinoma using a digital cholangioscopy to deliver the laser: A) cholangioscopic identification of the neoplastic stricture; B) placement of the lighting catheter across the neoplastic stricture; C) fluoroscopic control of the PDT catheter positioning; D) cholangioscopic appearance during lighting; E) repositioning of the fiber after the first lighting; F) final fluoroscopic control after the PDT session and plastic stenting.

After staging, the mass was deemed unresectable and the patient was referred for palliative treatment. After multidisciplinary meeting with the oncology team, was proposed an adjunct PDT to address the recurrent biliary obstruction. The procedure was uneventful and had duration of 150 min (Figure 3). At the end, was performed biliary drainage with a 10 Fr plastic stent, since self-expandable metallic stent was not available at that moment.

In the following week, the patient did not report any photosensitivity. There was, however, an early recurrence of the cholestasis complicated with cholangitis. The ERCP revealed obstruction of the stent with debris and biliary sludge secondary to tumor necrosis. Biliary sweeping followed by new drainage resolved the patient’s acute condition. Thereafter, he remained asymptomatic for three months, the longest period since the onset of the disease. At his next recurrence, he underwent drainage with a self-expandable metallic stent.

The patient kept on with systemic treatment and ultimately required duodenal stenting for palliation of dysphagia. He perished due to complications of lung metastases two years after the onset of symptoms and 15 months after the PDT session.

## DISCUSSION

This is the first report of a digital cholangioscopy-guided biliary PDT outside the USA. Moreover, the employment of a Brazilian laser source and the good clinical outcome make this report unique and relevant.

The PDT has been widely employed over the last decade for local specific treatment of unresectable biliary neoplasia. In fact, several studies have already evaluated the effectiveness of PDT plus biliary drainage and demonstrated the superiority of this association in comparison with sole drainage^3^ ^,^ ^9^ ^,^ ^18^. Also, some publications have demonstrated PDT plus chemotherapy to be superior to chemotherapy alone^13^. In this sense, Moole *et al* ^7^ recently published a meta-analysis pooling 10 studies and 402 patients comparing PDT plus biliary stenting vs. only biliary stenting. The authors demonstrated that patients undergoing adjunct PDT had a higher success rate of biliary drainage (defined as >50% decrease in bilirubin levels within seven days), increased survival rates, significant improvement in Karnofsky score, and lower rates of post-procedure cholangitis. Despite the high heterogeneity, a subgroup analysis involving only prospective studies confirmed all these findings except for the reduction in the rate of cholangitis^7^.

As the main side effect, photosensitivity occurs in about 11% of patients^7^. However, it can be effectively prevented with simple recommendations such as to avoid exposure to natural and artificial lights, and wearing sunglasses. Other factors that hampered the wide acceptance of PDT are high costs and limited availability of equipment and expertise^11^. The use of local technology may significantly reduce costs, allowing wider availability of equipment and ultimately helping to nurture local expertise. Such information emphasizes the importance of the present report.

Some studies have already described the use of cholangioscopy to target the delivery of light energy. In 2011, Talreja *et al* ^17^ published a retrospective cohort study reporting a significant reduction in the fluoroscopy exposure time of patients undergoing cholangioscopy-guided PDT compared to those undergoing standard PDT. Although the difference did not reach statistical significance, the authors also found improvement in overall survival: the intervention group (PDT + cholangioscopy) had an average increase of 186 days in survival.

Hypothetically, visual control of the laser delivery might optimize treatment, leading to increased tumor necrosis and less exposure of healthy tissue to light explaining the increase in mean survival found by Talreja *et al* ^17^. Of note, the authors employed the first generation cholangioscope. The second generation, named digital cholangioscope, carries significant improvement in both image quality and handling, which could further enhance the benefit of the association. To date, only one case of PDT with digital cholangioscopy has been reported^15^. The present study is, therefore, the second report in the world, the first outside the USA, and the only one to employ a locally-developed light source.

Our report is not exempt from limitations. The level of evidence is low and generalization of these results is not advisable. However, it can and should be used to support robust prospective studies that might refine the method and standardize the procedure. Moreover, our patient was not procedure-naïve. If he were, the outcome of the PDT might have been better once usually the earlier the treatment, the better the outcome. Finally, we did not have a self-expandable metallic stent for immediate drainage after the PDT session. The use of a plastic stent probably explains the early recurrence of the cholestasis, which could have been avoided if a larger metallic stent had been employed at first.

## CONCLUSION

Digital cholangioscopy-guided biliary PDT is feasible and seems safe and effective as an adjunct modality in the palliation of extrahepatic cholangiocarcinoma. 
